# *Terminalia arjuna* Switches from Adaptive to Survival Strategy Under Severe Water Stress

**DOI:** 10.3390/plants15060888

**Published:** 2026-03-12

**Authors:** Lumat Afrin Jui, Tahsin Chowdhury, Md. Ahosan Habib Ador, Rahela Khatun, Mohammed Masum Ul Haque, Biplob Dey, Romel Ahmed

**Affiliations:** 1 Department of Forestry and Environmental Science, Shahjalal University of Science and Technology, Sylhet 3114, Bangladesh; juilumat@gmail.com (L.A.J.); ador.forest@gmail.com (M.A.H.A.);; 2 Center for Research in Environment, iGen and Livelihood (CREGL), Sylhet 3114, Bangladesh; 3 Institute of Climate and Energy Systems: Troposphere (ICE-3), Forschungszentrum Jülich, 52428 Jülich, Germany; 4 Bioclimatology, Faculty of Forest Sciences and Forest Ecology, Georg-August-Universität Göttingen, Büsgenweg 2, 37077 Göttingen, Germany

**Keywords:** drought, plant stress, climate change, mineral content, water use efficiency, oxidative stress marker, stomata

## Abstract

*Terminalia arjuna* (Arjun) is a tropical deciduous tree species significantly valued for its pharmaceutical properties for various heart diseases, as well as its economic role in the sericulture industry. However, the growth performance and physiological responses of *T. arjuna* under water stress conditions remain largely unexplored, particularly in the context of increasing climate variability and the growing challenges posed by climate change. Therefore, this study aimed to examine the morpho-physio-biochemical alterations, nutrient uptake changes, and adaptive strategies under different degrees of water stress with respect to field capacity ($F_{wc}$), maintained at 100% $F_{wc}$ (control), 75% $F_{wc}$ (mild), 50% $F_{wc}$ (moderate), and 25% $F_{wc}$ (severe). Key growth parameters, including shoot and root length, leaf traits and shoot dry biomass, were significantly (*p* < 0.05) reduced under the given water stresses. Root dry biomass showed a distinct response, increasing under mild to moderate water stress but failing to sustain its levels under severe stress. Increasing drought severity resulted in a substantial reduction in stomatal density (15–37%), while stomatal size increased (18–49%) under mild to moderate stress but decreased under severe stress. These responses were associated with significant reductions in gas exchange traits (45–75%), whereas water use efficiency increased by 59–99%, reflecting a survival-focused adaptive mechanism. Moderate water stress triggered the stress responses in *T. arjuna* through high proline accumulation and increased oxidative stress markers. The most critical impact was found under the severe stress with a substantial reduction in leaf relative water content and membrane stability index (MSI), although MSI was sustained above the critical threshold, reflecting cellular protection. Increased stress intensity also altered mineral uptake, decreased major nutrients, and increased potassium and calcium content, indicating an adaptive strategy. These findings suggest a threshold effect, where *T. arjuna* tolerates mild stress well and activates adaptive morpho-physiological mechanisms under moderate stress but shifts to survival-focused strategies under severe stress. The demonstrated tolerance of *Terminalia arjuna* to mild–moderate drought suggests that climate-resilient forestry policies and conservation programs should prioritize its cultivation and restoration in drought-prone landscapes while ensuring adequate water management to prevent severe stress and sustain its medicinal and economic benefits.

## 1. Introduction

*Terminalia arjuna* (Arjun) is a tropical, fast-growing, deciduous forest tree species (Family: Combretaceae), native to the Indian subcontinent. For centuries, this species has been widely recognized as a traditional medicine, most significantly for its effectiveness in treating various heart diseases [1]. The bark of *T. arjuna* is a source of various bioactive compounds, rich in phenolics, flavonoids, glycosides, and tannins, which work against oxidation and reduce inflammation, as well as inhibit microbial infection [2]. Besides its medicinal importance, *T. arjuna* plays an important role in agroforestry, as it is widely used as a shade tree [3]. Additionally, it holds significant importance as a food source for the tasar silkworm (*Antheraea mylitta*), making it an economically important species, especially in India [4]. Given its wide-ranging significance in pharmaceutical, environmental, ecological, and economic contexts, ensuring proper regeneration and sustainable management is of fundamental importance [5].

Climate change has become the uttermost environmental challenge in the current period, impacting rainfall patterns and temperatures and intensifying the frequency of paramount weather events, which could transform the present habitat distribution and suitability and the phenology of many plant species and jeopardize their survival [6]. Drought is one of the most frequent abiotic stresses resulting from prolonged periods of inadequate precipitation or water availability caused by global climate change [7]. Water scarcity is progressively heightening concerns in drought-prone areas of Bangladesh, particularly in the northwestern regions, as a result of insufficient precipitation and excessive groundwater exploitation for irrigation purposes simultaneously combined with the detrimental impacts of climate change [8,9]. Plants undergo water stress whenever the availability of water to their roots is impeded or if the evapotranspiration rate gets excessively high, causing detrimental impacts on plants’ physio-biochemical and molecular processes [10,11].

Drought stress triggers certain morphological and physiological responses in plants, including the regulation of stomatal aperture, decline in photosynthetic pigments, disruption of gas exchange processes and alterations in phytohormone accumulation [12]. In response to water limitation, plants formulate strategies for stress tolerance by efficiently regulating the distribution of total assimilates during arid periods, maintaining an optimal balance between root and shoot growth [13]. Water deficit conditions also stimulate the production of oxidative molecules (ROS), such as hydrogen peroxide (H_2_O_2_) and superoxide anion (O_2_), which can cause cellular damage [14]. However, plants’ protection against oxidative stress is facilitated by enzymatic and non-enzymatic antioxidant systems, which hold a critical role in minimizing the detrimental impacts of reactive oxygen species (ROS) [11,15]. One of the mechanisms to acclimatize to drought stress is the accumulation of osmoprotectants such as proline, amino acids and soluble sugars, which contribute to osmotic adjustment and stabilize cell membranes along with various protein compounds [16]. Additionally, nutrient allocation in plant cells is closely linked with water availability; reduced soil moisture limits nutrient diffusion to root surfaces, thus restricting uptake and translocation to leaves [17,18]. Stomatal response is also influenced by nutrient redistribution; for example, potassium and calcium play a critical role in stomatal regulation by modulating guard cell turgor, thereby influencing photosynthesis and water use efficiency under stress conditions [19], although the response varies among plant species.

It is predicted that due to overharvesting and commercial demand along with climate change, high-value medicinal plants are under great risk of extinction [1]. In order to sustain productivity, it is vital to introduce drought-tolerant native plant species in drought-prone regions through the process of regeneration. Therefore, it is imperative to conduct thorough investigations into the response mechanisms of plants to water shortage to ascertain the sensitivity or resistance status of different species; medicinal plant species in particular are of significant concern. A study on the effects of drought stress and elevated temperature on *T. arjuna* showed adaptive physiological responses to stress, with improved water use efficiency [20]. Another study on *T. arjuna* seedlings under salt stress observed high germination sensitivity to salt stress and medium salt stress tolerance by altering several growth performances and maintaining water use efficiency [21]. Liu et al. [22] conducted research on *Salvia miltiorrhiza,* where they found that water stress decreased shoot and root dry weight with an increased root:shoot ratio and enhanced the most active constituents. In a separate study, *Tagetes minuta* reduced growth and photosynthetic pigments and increased oxidative stress markers, phenols, and enzyme activity while facing water stress [23].

Several studies have explored on how plants adapt to water deficiency by altering various functional and biochemical traits and uptaking macro-nutrients. Regarding the adaptive mechanisms of plants on root modification, osmotic adjustment, stomatal adaptations and nutrient reallocation under moderate and severe water stress, little is known about how *T. arjuna* responds to such conditions. Therefore, this study aimed to investigate the morpho-physiological and biochemical alterations in *T. arjuna* seedlings under different levels of water stress, along with a focus on nutrient uptake and translocation patterns.

## 2. Results

### 2.1. Effects on Morphological Parameters of T. arjuna

**Figure 1:**
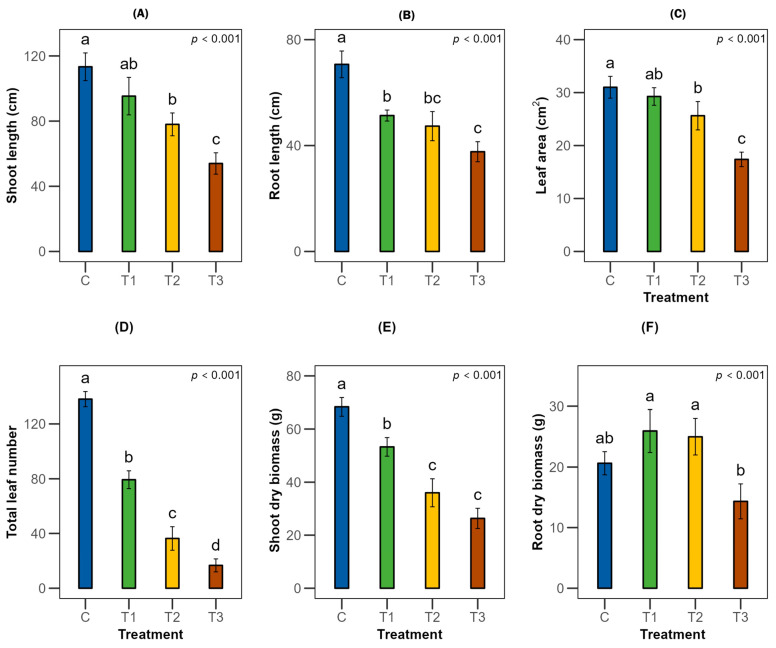
Effects of different stress levels on growth parameters (**A**) shoot length, (**B**) root length, (**C**) leaf area, (**D**) total leaf number, (**E**) shoot dry biomass, and (**F**) root dry biomass of *Terminalia arjuna* (*n* = 3). One-way ANOVA indicated significant differences with the corresponding *p*-values presented in the graphs. The different letter(s) in the same graph represent significant differences following Tukey’s post hoc test. [C, T1, T2, and T3 represent the control (100% $F_{wc})$, mild stress (75% $F_{wc}$), moderate stress (50% $F_{wc})$ and severe stress (25% $F_{wc}$), respectively]. Error bars represent the standard error of the mean (±SE).

**Figure 2:**
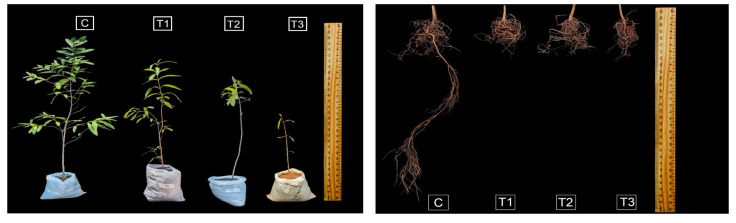
Effects of water stress on morphological parameters of *Terminalia arjuna*. [C, T1, T2, and T3 represent the control (100% $F_{wc})$, mild stress (75% $F_{wc}$), moderate stress (50% $F_{wc})$ and severe stress (25% $F_{wc}$), respectively].

Water stress treatments significantly influenced the growth parameters of *Terminalia arjuna* (*p* < 0.001), with the effects being more pronounced in the primary roots than in the shoots (Figure 1 and Figure 2). As irrigation levels decreased, *T. arjuna* showed a significant declining trend in shoot length, by 31% and 52% in moderate and severe water stress, respectively, relative to the control group. However, under mild stress (75% $F_{wc}$), shoot length was not statistically significant from the control (Figure 1A). Likewise, the highest mean primary root length (69 cm) was found in the control treatment (100% $F_{wc}$), while it decreased substantially by about 27% to 47% with increasing water scarcity (Figure 1B). Although leaf area showed no significant changes in mild stress, a 17% to 44% reduction was found under moderate and severe water stress (Figure 1C). Most importantly, leaves under the severe water stress started browning, and leaf shedding was observed after 30 days of treatment application, resulting in a significant decrease in total leaf number (43% to 88%) (Figure 1D). However, prolonged water deficiency also caused a substantial reduction in shoot dry biomass, ranging from 22% to 61% (Figure 1E). On the contrary, root dry biomass increased under mild to moderate stress but significantly decreased by one-third under severe water stress compared to the control (Figure 1F). The root:shoot ratio increased under mild, moderate, and severe water stress, with the most prominent increase found under moderate stress, with an approximately five-fold rise compared to the control (Figure S1).

### 2.2. Effects on Physiological Responses of T. arjuna

**Figure 3:**
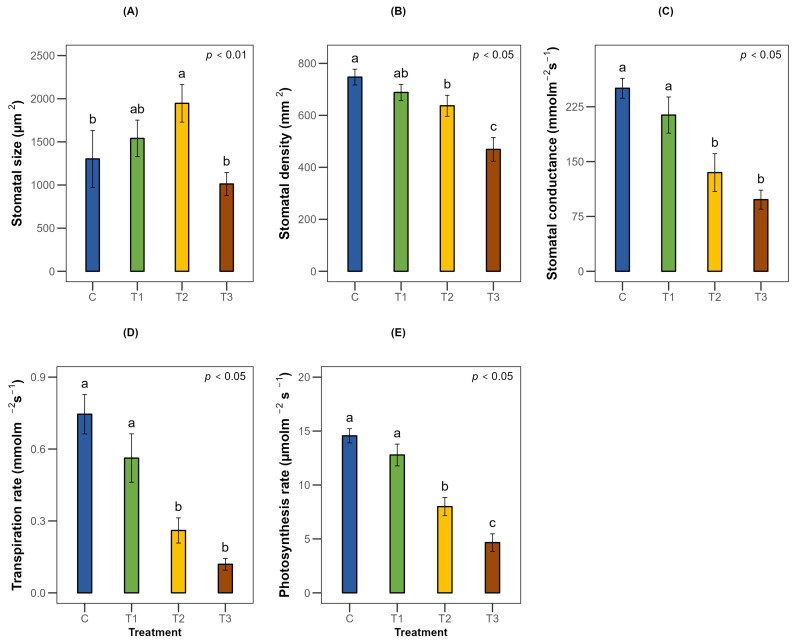
Effects of water stress on the physiological parameters (**A**) stomatal size, (**B**) stomatal density, (**C**) stomatal conductance, (**D**) transpiration rate and (**E**) photosynthesis rate of *Terminalia arjuna* (*n* = 3). One-way ANOVA indicated significant differences with the corresponding *p*-values presented in the graphs. The different letter(s) in the same graph represent significant differences following Tukey’s post hoc test. [C, T1, T2, and T3 represent the control (100% $F_{wc})$, mild stress (75% $F_{wc}$), moderate stress (50% $F_{wc})$ and severe stress (25% $F_{wc}$), respectively]. Error bars represent the standard error of the mean (±SE).

**Figure 4:**
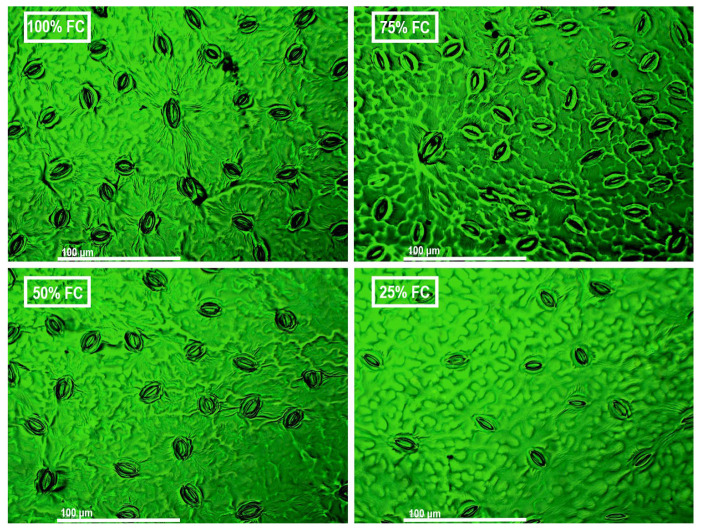
Microscopic images representing stomatal adjustments of *Terminalia arjuna* seedlings under different stress treatments. White scale bars represent 100 µm.

The physiological responses of *T. arjuna* seedlings under varying irrigation treatments differed significantly (*p* < 0.001). Stomatal size increased by 18% to 50% under mild and moderate water stress, whereas it decreased by 22% under severe stress relative to the control (Figure 3A and Figure S4). The highest stomatal density was found in the control seedlings, which was 747 mm^−2^. However, the density decreased by 15% and 37% under moderate and severe stress, respectively, while no significant changes were noted in the mild stress treatment (Figure 3B, Figure 4 and Figure S4). A similar trend was noticed for stomatal conductance, net photosynthetic rate and transpiration rate, all of which showed a significant decline by about one-half to two-thirds under 50% $F_{wc}$ and 25% $F_{wc}$ relative to the control (Figure 3C–E).

**Figure 5:**
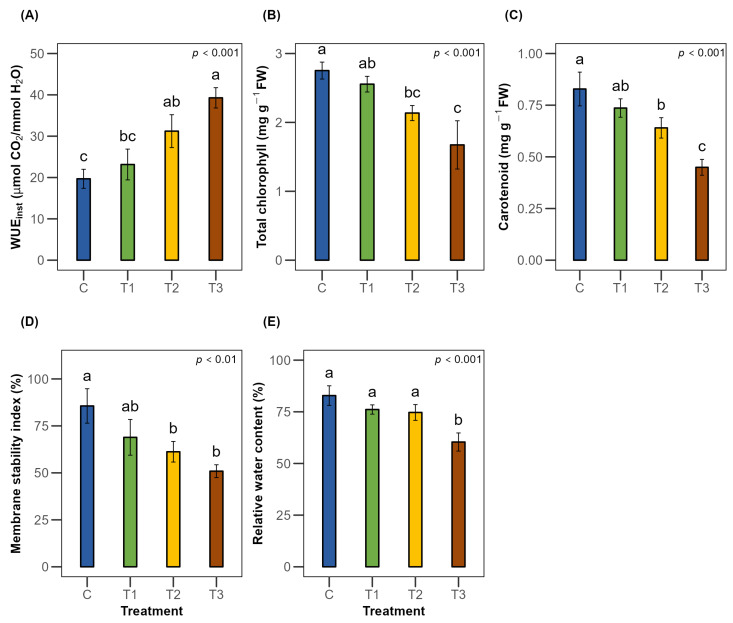
Effects of water stress on the physiological parameters (**A**) water use efficiency, (**B**) chlorophyll content, (**C**) carotenoid content, (**D**) membrane stability index and (**E**) relative water content of *Terminalia arjuna* (*n* = 3). One-way ANOVA indicated significant differences with the corresponding *p*-values presented in the graphs. The different letter(s) in the same graph represent significant differences following Tukey’s post hoc test. [C, T1, T2, and T3 represent the control (100% $F_{wc})$, mild stress (75% $F_{wc}$), moderate stress (50% $F_{wc})$ and severe stress (25% $F_{wc}$), respectively]. Error bars represent the standard error of the mean (±SE).

Water use efficiency showed the highest result while facing severe stress with an increment of about 59% and 99% under 50% $F_{wc}$ and 25% $F_{wc}$, respectively, which was significant relative to 100% $F_{wc}$. Nevertheless, the increase under 75% $F_{wc}$ was not found to be statistically different than that of full-irrigation seedlings (Figure 5A). Although only severe water stress caused a significant reduction in leaf RWC (Figure 5E), photosynthetic pigments declined substantially under varying irrigation regimes. Total chlorophyll and carotenoid content decreased by about one-third to nearly one-half under moderate and severe stress, compared to full irrigation (Figure 5B,C). Additionally, the membrane stability index (MSI) exhibited a similar pattern, with significant reductions of about 28% and 41% under irrigation treatments equivalent to 50% $F_{wc}$ and 25% $F_{wc}$, respectively (Figure 5D).

### 2.3. Effects on Biochemical Responses of T. arjuna

**Figure 6:**
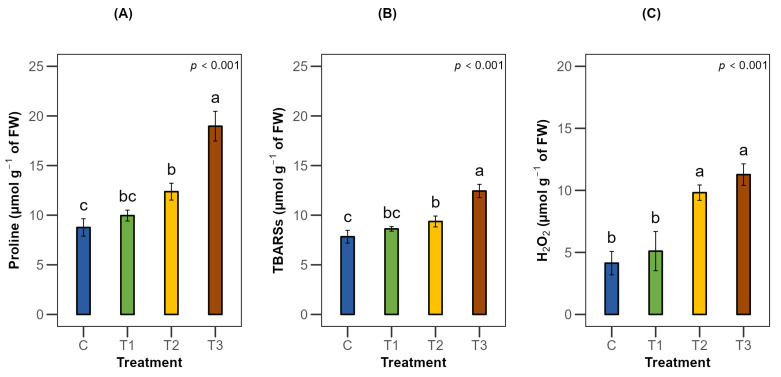
Effects of water stress on biochemical parameters (**A**) proline, (**B**) thiobarbituric acid reactive substances and (**C**) hydrogen peroxide of *Terminalia arjuna* (*n* = 3). One-way ANOVA indicated significant differences with the corresponding *p* values presented in the graphs. The different letter(s) in the same graph represent significant differences following Tukey’s post hoc test. [C, T1, T2, and T3 represent the control (100% $F_{wc})$, mild stress (75% $F_{wc}$), moderate stress (50% $F_{wc})$ and severe stress (25% $F_{wc}$), respectively]. Error bars represent the standard error of the mean (±SE).

Moderate (50% $F_{wc}$) and severe water stress (25% $F_{wc}$) induced biochemical defense responses while such responses were not significant under mild stress. Accumulation of proline, an osmoprotectant, increased by 42% and 117% under moderate and severe stress, respectively (Figure 6A). Lipid peroxidation markers, TBARSs, also increased by 20% under moderate stress and became more than double that (59%) under severe water stress, intensifying the cellular damage (Figure 6B). There was an elevated amount of ROS, such as H_2_O_2_ induction, which increased by two- to around three-fold with the progressive increase in water stress compared to full irrigation (Figure 6C).

### 2.4. Effects on Leaf Mineral Contents of T. arjuna

**Figure 7:**
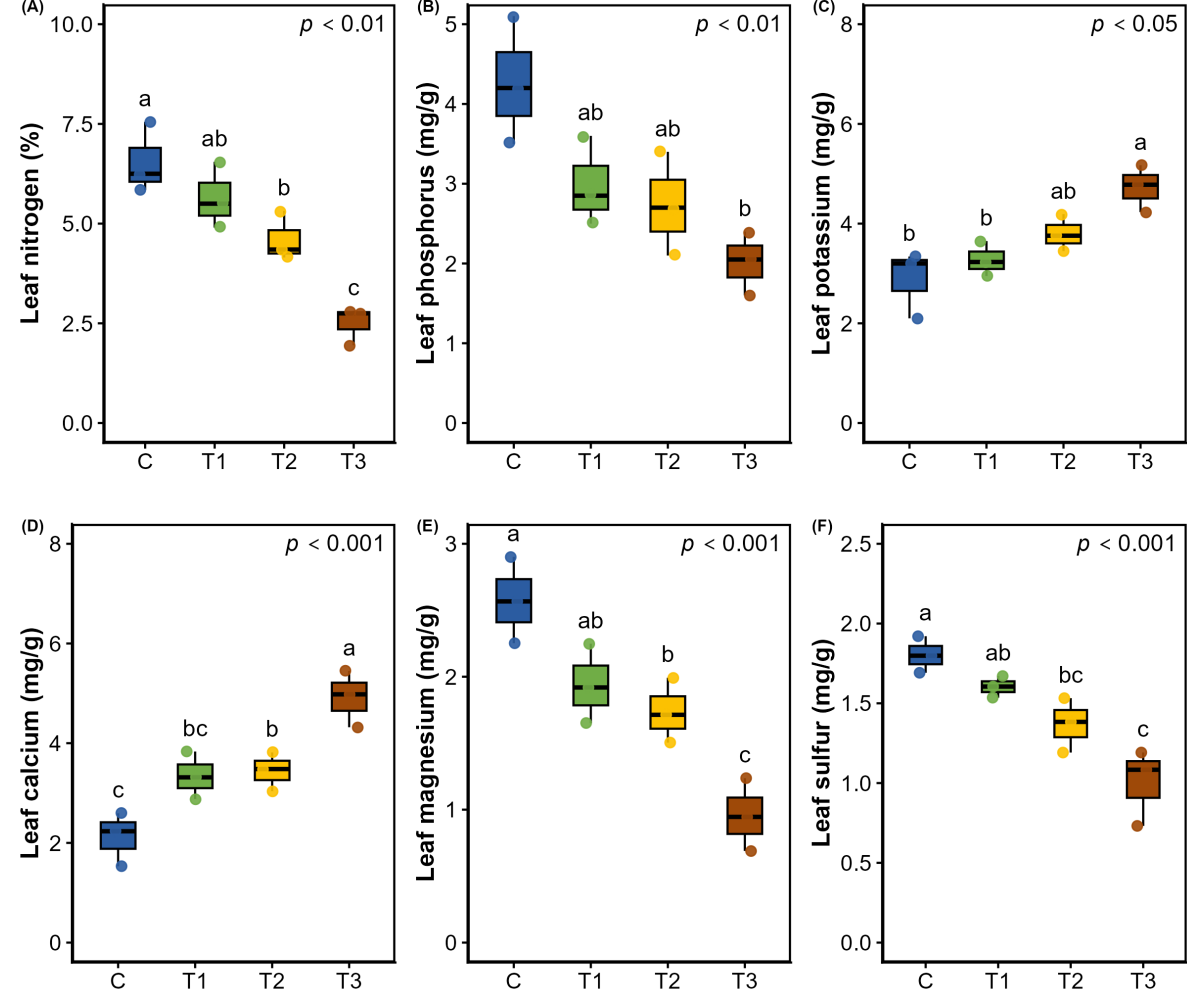
Effects of water stress on different mineral contents (**A**) leaf nitrogen, (**B**) leaf phosphorus, (**C**) leaf potassium, (**D**) leaf calcium, (**E**) leaf magnesium and (**F**) leaf sulfur of *Terminalia arjuna* (*n* = 3). One-way ANOVA indicated significant differences with the corresponding *p* values presented in the graphs. The different letter(s) in the same graph represent significant differences following Tukey’s post hoc test. [C, T1, T2, and T3 represent the control (100% $F_{wc})$, mild stress (75% $F_{wc}$), moderate stress (50% $F_{wc})$ and severe stress (25% $F_{wc}$), respectively]. Error bars represent the standard error of the mean (±SE).

Nutrient homeostasis was disrupted with increasing water stress except in the treatments where plants were irrigated at 75% field capacity (Figure 7 and Figure S2). Moderate and severe water stress differentially affected the acquisition and partitioning of nutrients. Interestingly, root nitrogen uptake increased under mild to moderate stress in comparison to the control, while it decreased significantly under severe stress (Figure S2A). In contrast, translocation of N content to the leaf was found to decrease with increasing stress intensity. Additionally, both root and leaf Mg and S contents also demonstrated a declining trend, whereas severe stress (25% $F_{wc}$) showed a marked reduction in phosphorus acquisition by about one-third relative to the control (100% $F_{wc}$) (Figure 7A–F and Figure S2B). A significant shift was found with increased accumulation of K and Ca in the root and translocation to leaves by about one-third to one-half under moderate to severe stress relative to the control (Figure 7C,D and Figure S2C,D).

### 2.5. Evaluation of Inter-Trait Relationships and Stress-Induced Variations in T. arjuna

**Figure 8:**
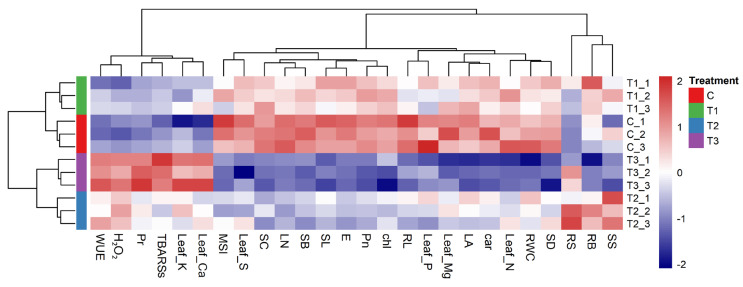
Heatmap analysis for varying levels of water stress treatments and morpho-physiological traits, biochemical parameters and mineral contents of *Terminalia arjuna* seedlings. [C, T1, T2, and T3 represent the control (100% $F_{wc})$, mild stress (75% $F_{wc}$), moderate stress (50% $F_{wc})$ and severe stress (25% $F_{wc}$), respectively]. SL: shoot length; RL: root length; LA: leaf area; LN: leaf number, SB: shoot dry biomass; RB: root dry biomass; RWC: relative water content; SC: stomatal conductance; SD: stomatal density; SS: stomatal size; Pn: photosynthesis rate; E: transpiration rate; WUE: water use efficiency; MSI: membrane stability index; chl: total chlorophyll; car: carotenoid; Pr: proline; TBARSs: thiobarbituric acid reactive substances; H_2_O_2_: hydrogen peroxide; RS: root:shoot ratio; Leaf_P: leaf phosphorus; Leaf_N: leaf nitrogen; Leaf_K: leaf potassium; Leaf_Ca: leaf calcium; Leaf_Mg: leaf magnesium; Leaf_S: leaf sulfur.

The Pearson’s correlation matrix demonstrated that all the growth parameters and biomasses were positively correlated with gas exchange traits and specific leaf nutrients (leaf N, P, Mg and S) (Figure S3). Interestingly, water use efficiency and leaf K and Ca contents exhibited a strong negative correlation with the morpho-physiological traits, suggesting an adaptive response with increasing stress intensity. The matrix also revealed that oxidative stress markers (H_2_O_2_ and TBARSs) negatively correlated with membrane stability and pigments, while showing a positive relation with proline accumulation (Figure S3). The clustered heatmap further validated these findings and revealed that severe water stress (T3) was different from the control, while the mild stress (T1) cluster was closer to the control than moderate and severe stress (Figure 8). In this cluster, *T. arjuna* maintained higher values for growth attributes, photosynthetic pigments, and gas exchange, indicating that mild stress caused very little effect. In contrast, the severe stress treatment formed a distinct cluster, with the highest levels of oxidative stress markers and proline and reserved water use efficiency, with the lowest physiological performances and reduced leaf mineral contents (leaf N, P, Mg, S). Interestingly, the notable increase in water use efficiency and the root:shoot ratio under T2 and T3 treatments underscore critical adaptive strategies to prioritize resource conservation and root development under water-deficit conditions.

## 3. Discussion

*T. arjuna* under varying water stresses triggered a set of altered morphological and physio-biochemical functions exhibiting both stress-induced damage and adaptive responses. The impeded growth functions include shoot and root length under the most stressful conditions compared to the control (Figure 1A,B and Figure 2). When irrigation was reduced to 50% field capacity equivalent, the shoot growth decreased to one-third of the control. Reduction in irrigation to 25% field capacity resulted in a decrease of about one-half in the shoot growth, suggesting the sensitivity of the species to moisture stress. The effect seems to be severe compared to other forest tree species, such as *Ficus benjamina* and *Conocarpus erectus* seedlings, which have a reduction in shoot length of up to 35% under low-irrigation regimes [24]. Limited water availability hinders the water mobility of plants (across the xylem to adjoining expanding cells), as water deficiency reduces the transpiration rate [25], which eventually suppresses stem growth. However, this reduction was associated with a decrease in primary root growth, even though root length was more affected than the shoot, which could be due to its immediate exposure to water stress. Although primary root length was most severely affected, possibly due to the slowdown of cell division and elongation, interestingly, root dry biomass increased under mild and moderate stresses. Consistently, increasing drought intensity substantially elevated the root:shoot ratio, with the most pronounced increase observed under moderate stress. This finding suggests a strategic reallocation of biomass from the shoot to root under water deficit. This response resulted from enhanced proliferation of secondary and tertiary roots, reflecting an adaptive mechanism of *T. arjuna* to enhance surface water acquisition. When plants face water stress, the accumulation of root abscisic acid (ABA) increases, which interacts with auxin signaling pathways by redistributing auxin from the primary root apex to the lateral root primordia, resulting in reduced primary root growth while inducing lateral root growth through a transcriptional network. However, severe stress may disrupt auxin transport, carbon limitation, and oxidative damage due to excessive ABA accumulation, which causes impaired cell division [26]. Thus, under severe stress, plants compromise overall root growth, suggesting the need for optimal moisture supply to sustain productivity.

Increasing drought severity altered nitrogen acquisition in roots and its translocation to leaves, thereby significantly affecting photosynthetic pigments and their function. The increment in root biomass is likely to be associated with increased root nitrogen content under 75% and 50% $F_{wc}$, by the plant partitioning less N in the leaf under stress conditions. Studies on *Moringa oleifera* under abiotic stresses also disclosed a similar outcome [27]. As most plant-available nitrogen is concentrated in the topsoil [28], the proliferation of secondary and tertiary roots under mild and moderate moisture stress is used for enhanced nitrogen uptake, indicating an adaptive strategy of this species to maintain plant functioning. In contrast, as water movement is essential for nutrient translocation from roots to other plant parts, limited water availability restricts upward water flow [25], resulting in the reduced N content in leaves. As N content is crucial for enhancing the metabolism of plants, its deficiency under limited water conditions affects chlorophyll synthesis, which ultimately reduces the plant’s ability to capture light [29]. This phenomenon contributes to the reduction in photosynthetic performance because photosynthesis heavily relies on light for essential functions. Related trends were also observed in other species, including *Helianthus annuus, Ficus benjamina* and *Conocarpus erectus* [24,30]. However, through this interconnected alteration process, the reduced photosynthetic rate ultimately limits plants’ ability to convert available water into yield under water-deficit conditions.

Nutrients essential for metabolic processes (phosphorus, magnesium, and sulfur) showed a declining trend with increasing drought severity, aligning with previous findings in *Moringa oleifera* species [27]. This reduction in P uptake and translocation illustrates an adaptive response. Since P is essential for the generation of ATP and NADPH, necessary for carbon fixation [31], this response can be interpreted as a strategy by which plants under severe water stress check CO_2_ fixation. Additionally, as S is vital for synthesizing necessary metabolites and mg is central to light harvesting through chlorophylls, their deficiencies contribute to the impairment of several physiological functions, including photosynthesis [32], which also impairs plant growth performances. Such a shift in nutrient allocation reflects a growth compensatory mechanism, focusing on survival over growth under drought conditions.

Water stress induces significant reductions in transpiration rate through alterations in leaf morphological traits and stomatal traits as well as Ca^2+^ regulated stomatal control. With increasing drought severity, a substantial decrease in transpiration rate was observed in *T. arjuna*. Several species like *Ziziphus rotundifolia* and *Moringa oleifera* also showed a declining pattern in transpiration rate and gas exchange under limited water availability [24,33]. These responses may be the result of lowering the formation of new leaves and accelerated senescence of flourished leaves [34]. In the present study, visible leaf senescence and abscission were observed after 30 days of severe stress, indicating a reduction in transpiration surface area. Stomatal traits also exhibited differential responses with increasing stress intensity. Under mild and moderate water stress, *T. arjuna* showed a substantial increase in stomatal size with decreasing stomatal density, which may act as a compensatory leaf anatomical adjustment to maintain CO_2_ diffusion and photosynthetic efficiency under limited water availability [35]. However, under severe water stress, the reduction in stomatal size may restrict the maximum stomatal opening, thus limiting water loss and improving stress resilience. This alteration strategy indicates that *T. arjuna* initially prioritized carbon assimilation under moderate stress but shifted toward water conservation under severe conditions.

Moreover, the reduction in transpiration rate is also linked with stomatal regulation, which is modulated by Ca^2+^ signaling. Interestingly, *T. arjuna* utilized this mechanism as an adaptive strategy by increasing leaf Ca content to maintain stomatal aperture. Notably, this response may be species-specific, as previous investigations reported both similar and contrasting findings in other species. *Cunninghamia lanceolata* and *Brassica napus* increased the uptake of Ca under water stress [36,37], whereas *Populus canadensis cv* and *Morus alba* showed a declining trend [38]. When plants experience water stress, it upregulates abscisic acid production, which induces an increase in Ca^2+^ levels in guard cells. This increment in Ca^2+^ activates specific ion channels which releases some ions from the guard cells and creates an osmotic imbalance, leading to water loss from the cells and resulting in turgor loss [39], hence causing stomatal closure. This is a stress response mechanism for adapting to drought that affects not only the closure of stomata but also a number of cellular activities, including membrane structure, and it maintains ion homeostasis and also triggers the regulation of resistance genes, thus facilitating the plant’s resilience against water shortage [40].

Despite all these drought-induced limitations, the species illustrated a significant increase in water use efficiency (WUE), rising by around 59% and 99% under moderate and severe stress. This increase in WUE was attributed to a differential reduction in transpiration relative to carbon assimilation, which might be caused by coordinated regulation of stomata. Even under severe water stress, limited stomatal aperture and residual photosynthetic capacity allowed the seedlings to maintain a higher ratio of carbon assimilation per unit water loss. Considering the other physiological performances like the substantial decline in relative water content (−27%) and pigments (22–46%) and elevated oxidative stress-induced damage, the higher WUE at this stress needs to be interpreted with reservation. Despite these impairments, *T. arjuna* relatively maintained carbon fixation while reducing water loss, hence increasing WUE. This phenomenon reflects a functional trade-off between carbon acquisition and water conservation rather than an indicator of improved plant performance. Thus, with increasing drought severity, the increase in WUE indicates a shift toward a survival-focused strategy by compromising overall physiological health.

The stress caused by water shortage accelerates the uptake of potassium to roots and translocation towards leaf parts in *T. arjuna.* This response may also be species-specific, as previous investigations reported contrasting findings in other species; for example, *Populus canadensis cv*, *Morus alba* and mycorrhizal pistachio showed a declining trend [38,41]. However, the internal requirement for K in plants under stress arises from prolonged ROS generation for the purpose of sustaining photosynthetic CO_2_ fixation or the stomatal opening [36]. Notably, increased K accumulation significantly contributes to higher WUE under increasing stress severity. As *T. arjuna* adopts a survival strategy rather than productivity, a certain amount of gas exchange is indispensable for photosynthesis and K^+^ plays a role by maintaining the stomatal opening, hence sustaining CO_2_ fixation. Additionally, *T. arjuna* increased the accumulation of root K and Ca while facing severe stress, which play a fundamental role in osmotic regulation, aiding the balance of root turgor, rigidity, and membrane integrity as well as enhancing water uptake efficiency [42], hence assisting the plants in coping with limited water availability.

Osmotic adjustment is an important strategy of plants subjected to water stress. In our study, *T. arjuna* reflected osmotic adjustment by accumulating proline under moderate and severe water stress treatments, consistent with the responses of *Ziziphus rotundifolia* and *Moringa oleifera* [27,33]. Proline serves multiple protective roles, like stabilizing membranes, scavenging reactive oxygen species (ROS), and maintaining turgor pressure by lowering osmotic potential [43,44]. Proline accumulation was parallel with the significant increase in oxidative stress markers (H_2_O_2_ and TBARSs). This oxidative stress caused cellular damage that was evidenced by the declining trend in membrane stability index (Figure 5D). Remarkably, under moderate to severe stress, *T. arjuna* illustrated a higher amount of proline accumulation, contributing to reduced cellular damage and maintaining membrane stability at least above the critical threshold. This response indicates *T. arjuna* prioritized cellular-level protection under severe water stress.

## 4. Materials and Methods

### 4.1. Experimental Design

The experiment was performed at the nursery of the Department of Forestry and Environmental Science, Shahjalal University of Science & Technology (SUST), Sylhet, Bangladesh (24°55′15″ N, 91°50′05″ E). The experiment was conducted in a naturally ventilated polyhouse from October 2022 to March 2023, and the average minimum temperature of the polyhouse was 15.3 °C and the maximum temperature was 36.5 °C, with a relative humidity range between 50% and 60% during the experimental period.

For germination, *Terminalia arjuna* seeds were collected from the SUST campus and then sterilized with 5% NaOCl (sodium hypochlorite) for 3–5 min to reduce microbial infection. After that, they were treated with 70% ethanol for around one minute followed by rinsing four times (each time for 1–2 min) with distilled water. Then, the seeds were soaked in an autoclave with distilled water for up to 24 h to expedite the germination process. After the completion of the seed treatment process, they were evenly sown in the prepared seed bed. After eight weeks of seed germination, the seedings were transplanted to polybags (10 cm × 13 cm) and kept 6 months for growth. Later, uniform-sized healthy seedlings were transferred to the nursery polybags (21 cm × 13 cm) comprising 4 kg of loamy soil with a p^H^ of 6.6 and an electrical conductivity of 1.13 ds/m mixed with manure in a ratio of 3:1. Then the seedlings were irrigated daily, maintaining 100% $F_{wc}$ for four weeks to normalize their growth. After completing the normalization period, the seedlings were arranged following a completely randomized design with four water treatment groups and three separate replications for each treatment. Thus, the total sampling number of the experiment was 12. The four different water treatments were then imposed, maintaining the following field capacity levels: control (100% $F_{wc}$), mild stress (75% $F_{wc}$), moderate stress (50% $F_{wc}$), and severe stress (25% $F_{wc}$). Soil moisture content at 100% $F_{wc}$ ($\theta_{w}$) was calculated by the soil weight (SW) with Formula (1). The soil moisture levels for the other three field capacities were then calculated based on the reference weight for 100% $F_{wc}$.

(1) $$\theta_{w}=\frac{{SW}_{saturated}\;-\;{SW}_{dry}}{{SW}_{dry}}\times 100\;$$

**Figure 9:**
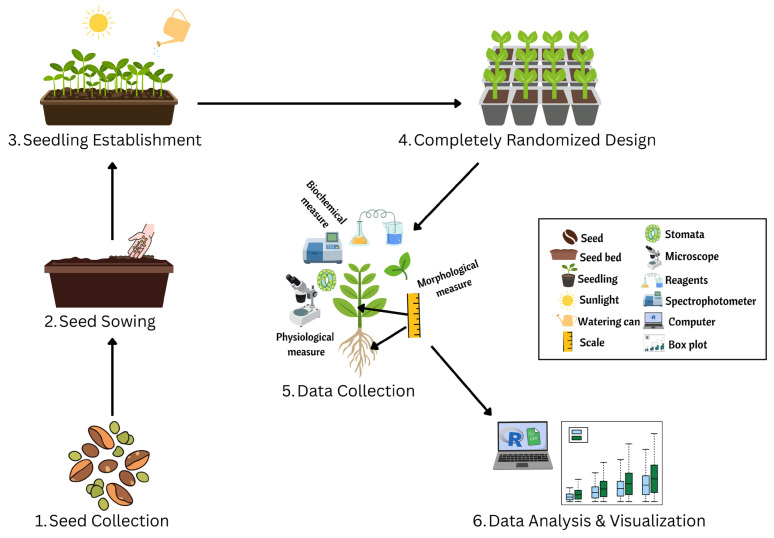
Schematic diagram of the experimental methodology, illustrating the workflow from seed collection to seedling establishment, experimental design, data collection and visualization.

To maintain consistent water stress throughout the experiment, the four polybags contained equally moist soil were placed in each of the block’s four corners during the initial setting of the poly bag experiment. The stress level was maintained by replenishing soil moisture by adding water to seedlings every day in an amount equal to the average amount of weight lost from these polybags through evapotranspiration. The experiment was carried out over 6 months with continuous observation and data collection throughout the study period, Figure 9.

### 4.2. Data Collection

The seedlings were harvested after 6 months of applying treatments. All morphological traits and macro-nutrients were measured after harvesting. Physiological and biochemical traits were measured in the last week of the experiment. To minimize variability, all physiological and biochemical traits were taken from three replicates. Each measurement was obtained using the top three mature leaves collected from individual seedlings.

#### 4.2.1. Morphological Responses of the Seedlings

The shoot and root length of the seedlings were determined with a wooden meter scale and the leaf area was estimated using a CI-202 Portable Laser Leaf Area Meter (CID Bio-Science, Inc., Camas, WA, USA). Both mature and new leaves were counted from each seedling to assess leaf number variability under different irrigation regimes. After completing the measurement, the harvested seedlings were dried using an oven drier at 70 °C for 72 h. Then using a digital electronic balance, the root dry biomass and shoot dry biomass of the seedlings were measured. Furthermore, the root:shoot ratio was calculated dividing root dry biomass by shoot dry biomass for each seedling.

#### 4.2.2. Physiological Responses of the Seedlings

For determining stomatal conductance, a portable leaf porometer (AP4, Delta-T Devices, UK) was used, and the net photosynthetic rate and transpiration rates of each sample were recorded with a handheld photosynthesis instrument (CI-340, CID Bio-Science, Inc., USA) at the time of 09:00–12:00 am. All of these three traits were taken on a sunny day with an ambient temperature of 30 to 36 °C and a photosynthetically active radiation (PAR) range of 550–580 μmol.m^−2^. s^−1^ at the time of 09:00–12:00 am to ensure accurate results. Intrinsic water use efficiency was calculated by dividing the net photosynthetic rate by the transpiration rate [45], where relative water content was calculated according to [46]. Leaf chlorophyll and carotenoid contents were measured using 80% acetone following the method given by [47]. A total of 100 mg of fresh leaf samples was taken from the top three mature leaves of each seedling and sterilized with 70% ethanol and distilled water. Afterwards, they were cut into small pieces and stored in the dark with 80% acetone (10 mL for each sample). After 3 days, quantification of the solution was performed by spectrophotometry at 480, 510, 645 and 663 nm. The total chlorophyll and carotenoid contents were then calculated following Formulas (2) and (3).

(2) $$Total\;chlorophyll\;(mg.\;g^{-1}\;FW)\;=\;\frac{\left\{20.2\;\left(A645\right)+\;8.02\;\left(A663\right)\right\}\;\times \;V}{1000\;\times \;W}$$

(3) $$Carotenoid\;content\;(mg.\;g^{-1}\;FW)\;=\;\frac{\left\{7.6\;\left(A480\right)+\;8.02\;\left(A510\right)\right\}\;\times \;V}{1000\;\times \;W}$$

For calculating the stomatal density, a transparent nail paint was applied to make a representation of the leaf abaxial surface according to [48]. Then, the cast was detached using transparent tape and positioned on a microscope slide. After that, the slides were placed on a light microscope (Motic Panthera C2 Trinocular, Motic Asia, Hong Kong, China), and leaf stomata were taken with a snap at 40× magnification. One imprint was taken from each leaf, with one leaf sampled per seedling, resulting in three samples from each treatment group. Furthermore, the density of the stomata was calculated with ImageJ (Version 1.54j, National Institute of Health, USA) by manually counting the number of stomata within a uniform size of field (~0.061 mm^2^), and the values were expressed as the number of stomata per mm^2^. Stomatal size was also quantified using the ImageJ after calibrating the images with a micrometer scale. The stomatal length and width of all clearly visible stomata from each image were measured and the stomatal area was further calculated. The mean stomatal size for each image was expressed in µm^2^ units.

In order to determine the membrane stability index (MSI), at first, 100 mg of fresh leaf samples, excluding the midribs, was sectioned into minute pieces and put into a test tube with 10 mL of distilled water. Afterwards, using a water bath at 40 °C, the samples were heated (30 min). After cooling down, the first electric conductivity (EC1) value was determined using a bench-top p^H^ meter (Model: HI5522, Hanna Instrument, Laval, QC, Canada). Later, a second electric conductivity (EC2) value was measured after heating at 100 °C (10 min). The MSI was then calculated following Formula (4) [49].

(4) $$MSI\left(\%\right)=1-\frac{EC1}{\mathrm{EC}2}\times 100$$

#### 4.2.3. Biochemical Response Traits and Nutrient Accumulation

Leaf proline content was measured using the acid ninhydrin method given by [50]. Approximately 0.5 g of dry leaf samples was homogenized with a solution of 3% sulfosalicylic acid (10 mL) and then filtered using Whatman filter paper. Afterward, in the test tube, the sample extract (2 mL) was mixed with acid ninhydrin and glacial acetic acid (2 mL) and then incubated using a water bath (100 °C; 1 h). After cooling the samples in an ice bath, the chromophore-containing toluene (4 mL) was separated from the liquid, and the absorbance was measured at 520 nm using a UV–VIS spectrophotometer (UV-1800, Shimadzu Corporation, Kyoto, Japan).

Following [51], the thiobarbituric acid reactive substances (TBARSs) were evaluated using thiobarbituric acid (TBA). A total of 0.5 g dry leaf was homogenized with 5 mL trichloroacetic acid (TCA) (0.1%) and centrifuged (10,000) for 10 min. After that, a 4 mL solution mixture containing 20% TCA and 0.5% TBA was mixed with 1 mL of supernatant. The mixture was heated for 15 min (95 °C) using a water bath and further cooled using an ice bath. The solution was again centrifuged and the absorbance of the final supernatant was estimated at 532 nm.

Leaf hydrogen peroxide (H_2_O_2_) accumulation was measured following the potassium iodide (KI) method formulated by [52]. For this, 0.5 g of dry leaf samples was homogenized with 0.1% TCA (3 mL) and centrifuged at 12,000 rpm for 15 min. The reaction mixture was prepared with the supernatant (0.5 mL), 1M KI (1 mL) and 0.5 mM Potassium Phosphate buffer (p^H^ 7.0) (0.5 mL). The absorbance was taken at 390 nm.

For evaluating root and leaf macro-nutrients (nitrogen (N), magnesium (Mg), sulfur (S), potassium (K), calcium (Ca) and phosphorous (P)) under varying degrees of water stress, 0.25 g of dried root and leaf samples was digested with HNO_3_-H_2_O_2_ solution with a volume ratio of 7:1 using a closed digestion system. After digestion, the solution was diluted up to 100 mL. Then the concentration of macro-nutrients was estimated using Inductively Coupled Plasma Optical Emission Spectroscopy (ICP-OES, iCAP PRO Series, Bermen, Germany). For measuring the root and leaf N content, the samples were digested with H_2_SO_4_ and H_2_O_2_ and then the amount was measured using an automatic Kjeldahl analyzer following the Kjeldahl method given by [53].

### 4.3. Data Analysis

The data analysis was conducted using R in RStudio (R version 4.4.0) with the following library packages: ggplot2, ggpubr, multicompView, car, dplyr, ggthemes, pheatmap, tidyr, Hmisc and corrplot. Initially, data normality was checked using the Shapiro–Wilk test and homogeneity of variance using Levene’s test. One-way analysis of variance (ANOVA) was done to address the statistically significant differences in the response traits of *T. arjuna* between each treatment group. Tukey’s post hoc test was applied at a 95% confidence interval to further identify the significant differences between different stress levels. Correlation coefficients were evaluated using Pearson’s correlation matrix to illustrate the relationships between morpho-physiological traits, biochemical parameters and mineral contents of *T. arjuna* seedlings under varying levels of water stress. A heatmap was created to visualize the variations in morpho-physio-biochemical traits and nutrient contents across different water stress treatments through a gradient color scale.

## 5. Conclusions

This extensive study demonstrates the responses of *Terminalia arjuna* to varying levels of water stress through a combination of morpho-physiological and biochemical alterations, as well as nutrient redistribution. Under drought conditions, growth attributes declined significantly with increasing root:shoot ratio, reflecting both stress-induced damage and adaptive strategies. The elevated acquisition of potassium and calcium, both recognized as signaling molecules, suggests their significant role in critical stomatal regulation and CO_2_ fixation. Notably, water use efficiency (WUE) improved with increasing stress severity, reflecting a growth compensatory mechanism, though this came at the cost of transpiration and photosynthetic efficiency, resulting in reduced productivity. Increased accumulation of TBARSs and H_2_O_2_ under water stress indicated oxidative damage, while elevated levels of proline confirmed osmotic adjustment. Importantly, a higher accumulation of proline, particularly under severe drought, contributed to cellular protection, thus maintaining membrane stability above the critical threshold level. However, despite some capacity for short-term acclimation, *T. arjuna* showed vulnerability to severe stress, shifting toward survival-oriented strategies by compromising growth, indicating that optimal water supply is necessary for sustaining productivity. Further studies should focus on wood anatomical responses and antioxidant defense mechanisms as well as investigating post-stress recovery processes to gain a deeper understanding of the resilience potential of *T. arjuna*. Additionally, gene expression analysis would help identify drought-responsive genes, enabling the development of stress tolerance markers for this medicinally and ecologically valuable native species. This study highlights the species’ moderate drought tolerance and the potential role of ion regulation, stomatal adjustment, and oxidative responses in enhancing drought resilience in *T. arjuna* seedlings. Therefore, a clear understanding of these adaptive strategies could inform efficient nursery practices and guide the selection and management of *T. arjuna* in afforestation and conservation programs, thereby supporting sustained productivity, particularly in drought-prone ecosystems.

## Data Availability

Data will be provided upon a request to the corresponding author.

## Supplementary Materials

The following supporting information can be downloaded at: https://www.mdpi.com/article/10.3390/plants15060888/s1.
