# Supplementary material for: Terminalia arjuna Switches from Adaptive to Survival Strategy Under Severe Water Stress
Source: Plants (Basel). 2026 Mar 12;15(6):888. doi: 10.3390/plants15060888 (PMC13029430; doi:10.3390/plants15060888)
Supplement: Supplementary file 1 [file plants-15-00888-s001.zip › plants-4178508-supplementary.pdf]

Supplementary Information

Terminalia arjuna Switches from Adaptive to Survival Strategy under Severe Water Stress

Lumat Afrin Jui 1, Tahsin Chowdhury 1, Md. Ahosan Habib Ador 1,2, Rahela Khatun 1, Mohammed Masum Ul Haque 1, Biplob Dey 2,3,4* and Romel Ahmed 1,2, *

[1]Department of Forestry and Environmental Science, Shahjalal University of Science and Technology, Sylhet 3114, Bangladesh
[2]Center for Research in Environment, iGen and Livelihood (CREGL), Sylhet 3114, Bangladesh
[3]Institute of Climate and Energy Systems: Troposphere (ICE-3), Forschungszentrum Jülich, 52428 Jülich, Germany
[4] Bioclimatology, Faculty of Forest Sciences and Forest Ecology, Georg-August-Universität Göttingen, Büsgenweg 2, 37077 Göttingen, Germany
* Correspondence: biplobforestry@gmail.com (B.D.); romel-fes@sust.edu (R.A.)

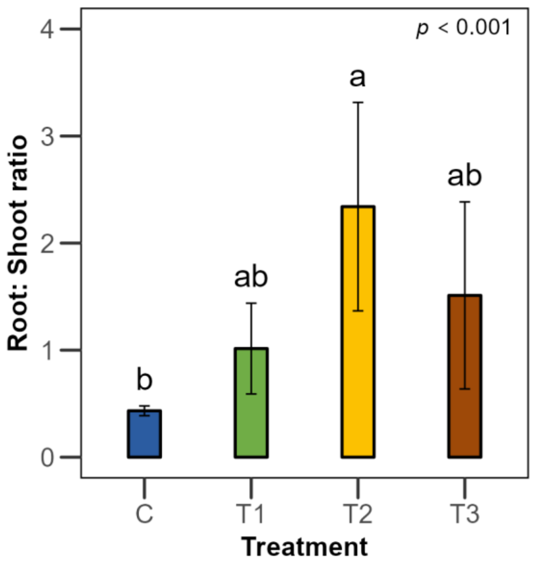

Supplementary Figure S1: Effects of water stress on root: shoot ratio of *Terminalia arjuna* (n=3). One-way ANOVA indicated significant differences with the corresponding p values presented in the graphs. The different letter(s) in the same graph represent significant differences following Tukey's post hoc test. [C, T1, T2, T3 represents the control (100% F_{wc}), mild-stress (75% F_{wc}), moderate-stress (50% F_{wc}) and severe-stress (25% F_{wc}), respectively]. Error bars represented the standard error of the mean (±SE).

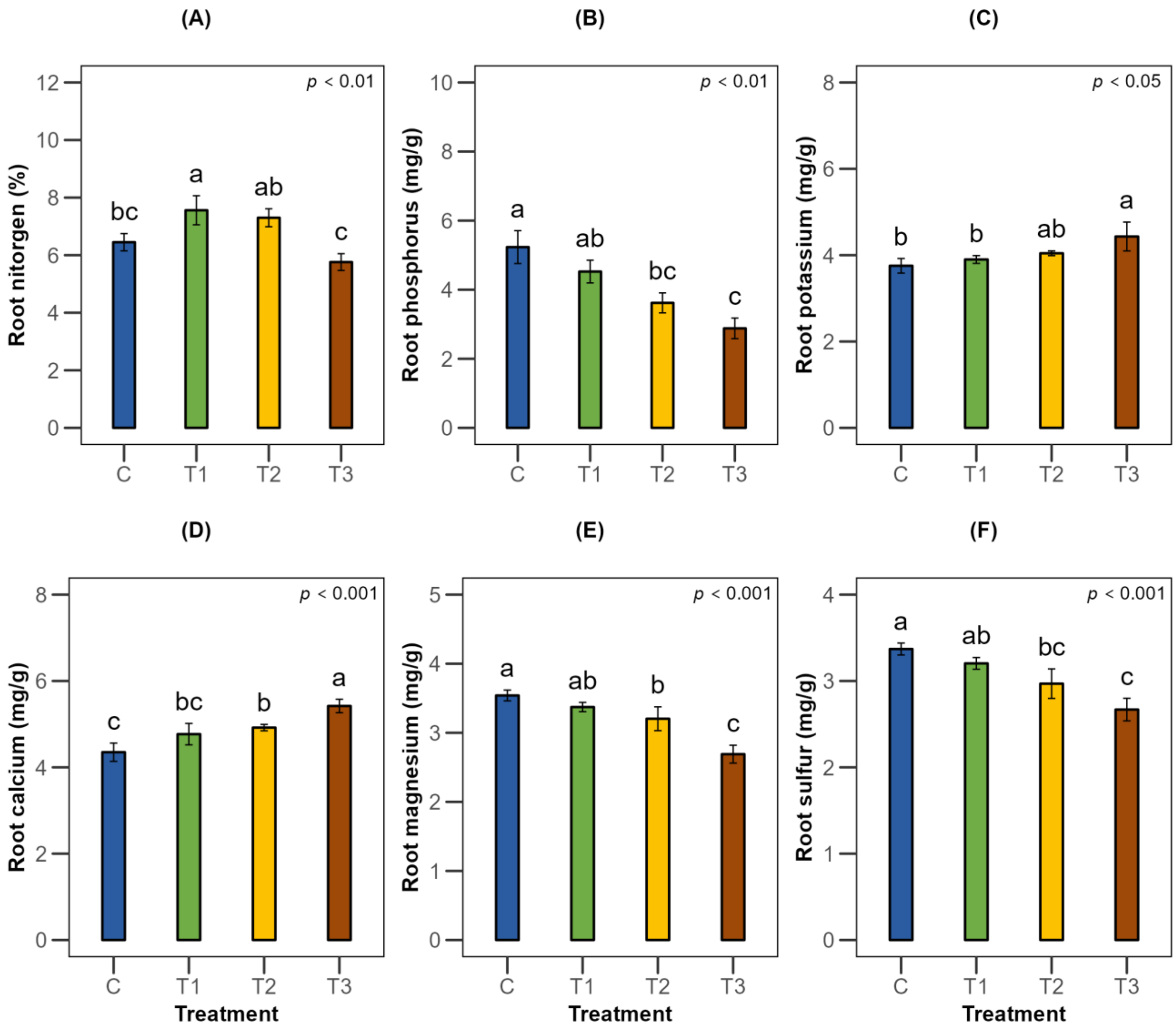

Supplementary Figure S2: Effects of water stress on root mineral contents of *Terminalia arjuna* (n=3). One-way ANOVA indicated significant differences with the corresponding p values presented in the graphs. The different letter(s) in the same graph represent significant differences following Tukey's post hoc test. [C, T1, T2, T3 represents the control (100% $\boldsymbol{F_{wc}}$), mild-stress (75% $\boldsymbol{F_{wc}}$), moderate-stress (50% $\boldsymbol{F_{wc}}$) and severe-stress (25% $\boldsymbol{F_{wc}}$), respectively]. Error bars represented the standard error of the mean (±SE).

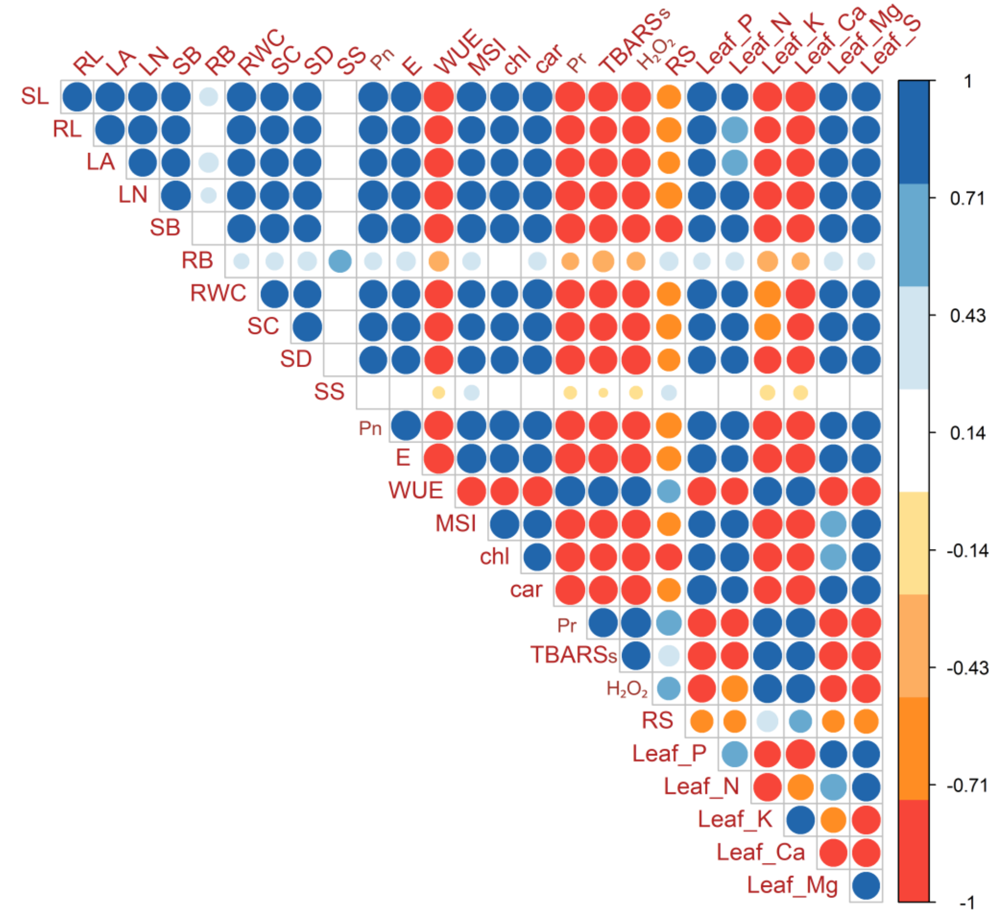

Supplementary Figure S3: Pearson's correlation matrix showing relationships between morpho-physiological traits, biochemical parameters and mineral contents of *Terminalia arjuna* seedlings under varying water stresses (100% $\boldsymbol{F_{wc}}$, 75% $\boldsymbol{F_{wc}}$, 50% $\boldsymbol{F_{wc}}$, 25% $\boldsymbol{F_{wc}}$). Circle color and size indicate the strength and direction of correlation. Large circles denote strong relationships and smaller circles denote weaker relationships. The color scale on the right ranges from −1 (strong negative correlation) in dark red to +1 (strong positive correlation) in dark blue. Blank blocks indicated the non-significant correlation. SL: Shoot length; RL: Root length; LA: Leaf area; LN: Leaf number, SB: Shoot dry biomass; RB: Root dry biomass; RWC: Relative water content; SC: Stomatal conductance; SD: Stomatal density; SS: Stomatal size; Pn: Photosynthesis rate; E: Transpiration rate; WUE: Water use efficiency; MSI: Membrane stability index; chl: Total chlorophyll; car: Carotenoid; Pr: Proline; TBARSs: Thiobarbituric acid reactive substances; H_2O_2: Hydrogen peroxide; RS: Root: shoot ratio; Leaf_P: Leaf phosphorus; Leaf_N: Leaf nitrogen; Leaf_K: Leaf potassium; Leaf_Ca: Leaf calcium; Leaf_Mg: Leaf magnesium; Leaf_S: Leaf sulfur.

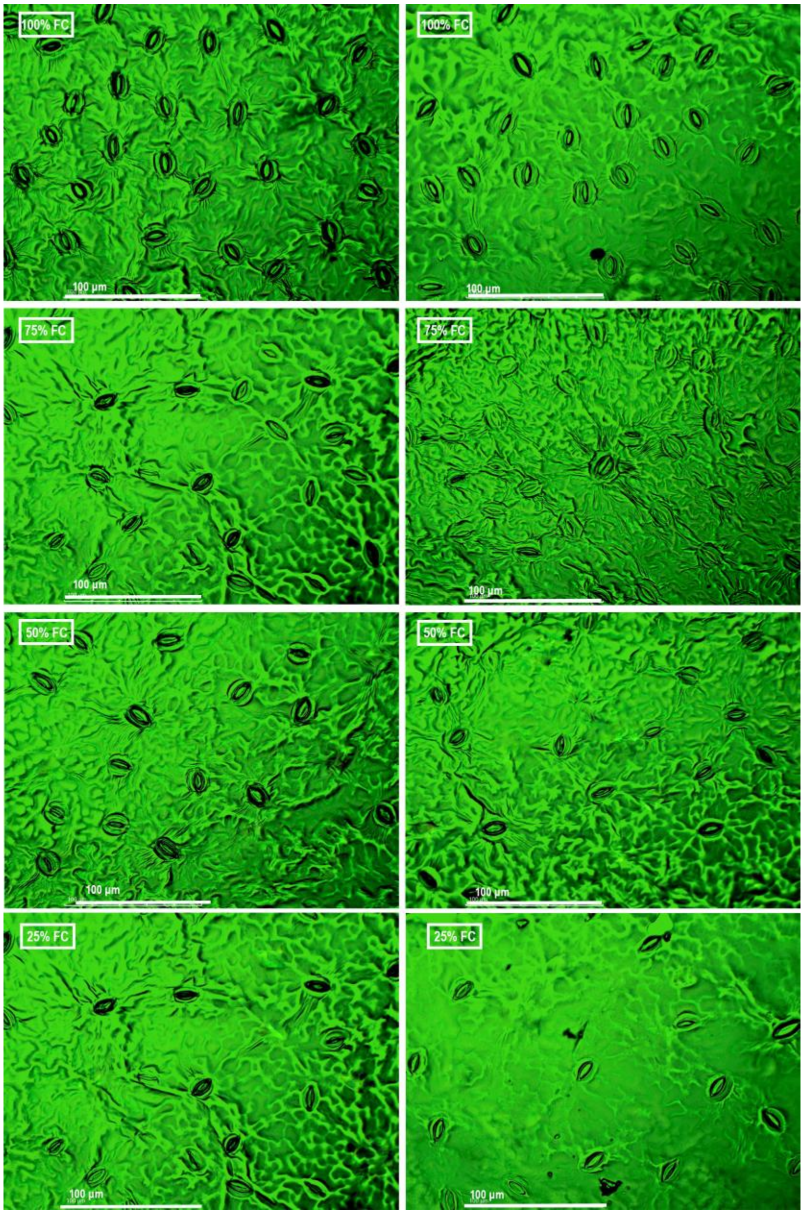

Supplementary Figure S4: Microscopic images represented stomatal adjustments of *Terminalia arjuna* seedling under different stress treatments. White scale bars represented 100 µm.

Supplementary Table: Analysis of Variance (ANOVA) for all the parameters:

Table-S1: Shoot Length

**Source**	**Df** (Degrees of freedom)	**Sum Sq**	**Mean Sq**	**F value**	**Pr(>F)**
**Treatment**	3	5758	1919.4	25.88	0.00018***
**Residuals**	8	593	74.2		

Table-S2: Root Length

**Source**	**Df**	**Sum Sq**	**Mean Sq**	**F value**	**Pr(>F)**
**Treatment**	3	1727.6	575.9	30.99	0.0000939***
**Residuals**	8	148.7	18.6		

Table-S3: Leaf Area

**Source**	**Df**	**Sum Sq**	**Mean Sq**	**F value**	**Pr(>F)**
**Treatment**	3	329.7	109.91	27.39	0.000147***
**Residuals**	8	32.1	4.01		

Table-S4: Total Leaf Number

**Source**	**Df**	**Sum Sq**	**Mean Sq**	**F value**	**Pr(>F)**
**Treatment**	3	25,097	8,352	296.52	0.000000068***
**Residuals**	8	340	42.50		

Table-S5: Shoot Dry Biomass

**Source**	**Df**	**Sum Sq**	**Mean Sq**	**F value**	**Pr(>F)**
**Treatment**	3	3118.0	1039.3	61.82	0.00000708***
**Residuals**	8	134.5	16.8		

Table-S6: Root Dry Biomass

**Source**	**Df**	**Sum Sq**	**Mean Sq**	**F value**	**Pr(>F)**
**Treatment**	3	252.04	84.01	10.06	0.00433 **
**Residuals**	8	66.82	8.35		

Table-S7: Stomatal Size

Source	Df	Sum Sq	Mean Sq	F value	Pr(>F)
**Treatment**	3	1,405,225.03	468,408.34	8.55	0.0071 **
**Residuals**	8	438,274.21	54,784.28		

Table-S8: Stomatal Density

Source	Df	Sum Sq	Mean Sq	F value	Pr(>F)
**Treatment**	3	128846	42949	30.66	0.0000975***
**Residuals**	8	11205	1401		

Table-S9: Stomatal Conductance

Source	Df	Sum Sq	Mean Sq	F value	Pr(>F)
**Treatment**	3	44091	14697	35.98	0.0000542***
**Residuals**	8	3267	408		

Table-S10: Transpiration Rate

Source	Df	Sum Sq	Mean Sq	F value	Pr(>F)
**Treatment**	3	0.7259	0.24198	47.58	0.0000191***
**Residuals**	8	0.0407	0.00509		

Table-S11: Photosynthesis Rate

Source	Df	Sum Sq	Mean Sq	F value	Pr(>F)
**Treatment**	3	170.811	56.937	80.97	0.000002***
**Residuals**	8	5.626	0.703		

Table-S12: Water Use Efficiency

Source	Df	Sum Sq	Mean Sq	F value	Pr(>F)
**Treatment**	3	689.3	229.75	22.39	0.000302***
**Residuals**	8	82.1	10.26		

Table-S13: Total Chlorophyll

Source	Df	Sum Sq	Mean Sq	F value	Pr(>F)
**Treatment**	3	2.0633	0.6878	16.91	0.000802***
**Residuals**	8	0.3254	0.0407		

Table-S14: Carotenoid

Source	Df	Sum Sq	Mean Sq	F value	Pr(>F)
**Treatment**	3	0.23654	0.07885	25.11	0.000201***
**Residuals**	8	0.02512	0.00314		

Table-S15: Membrane Stability Index

Source	Df	Sum Sq	Mean Sq	F value	Pr(>F)
**Treatment**	3	1926.06	642.02	11.91	0.00254**
**Residuals**	8	431.14	53.89		

Table-S16: Relative Water Content

Source	Df	Sum Sq	Mean Sq	F value	Pr(>F)
**Treatment**	3	803.4	267.79	17.46	0.000716***
**Residuals**	8	122.7	15.33		

Table-S17: Proline

Source	Df	Sum Sq	Mean Sq	F value	Pr(>F)
**Treatment**	3	196.3813	65.4604	61.94	0.000003***
**Residuals**	8	8.4552	1.0569		

Table-S18: TBARSs

Source	Df	Sum Sq	Mean Sq	F value	Pr(>F)
**Treatment**	3	43.4682	14.4894	35.09	0.000042***
**Residuals**	8	3.3032	0.4129		

Table-S19: H_2O_2

Source	Df	Sum Sq	Mean Sq	F value	Pr(>F)
**Treatment**	3	109.8961	36.6320	32.40	0.00012***
**Residuals**	8	9.0461	1.1308		

Table-S20: Leaf Nitrogen

Source	Df	Sum Sq	Mean Sq	F value	Pr(>F)
**Treatment**	3	27.282	9.094	17.24	0.000748***
**Residuals**	8	4.219	0.527		

Table-S21: Leaf Phosphorus

Source	Df	Sum Sq	Mean Sq	F value	Pr(>F)
**Treatment**	3	7.928	2.6428	6.85	0.0134*
**Residuals**	8	3.087	0.3858		

Table-S22: Leaf Potassium

Source	Df	Sum Sq	Mean Sq	F value	Pr(>F)
**Treatment**	3	5.736	1.9119	8.08	0.00835**
**Residuals**	8	1.893	0.2366		

Table-S23: Leaf Calcium

Source	Df	Sum Sq	Mean Sq	F value	Pr(>F)
**Treatment**	3	11.792	3.931	15.84	0.00997***
**Residuals**	8	1.985	0.248		

Table-S24: Leaf Magnesium

Source	Df	Sum Sq	Mean Sq	F value	Pr(>F)
**Treatment**	3	3.997	1.3322	16.16	0.000933***

**Residuals**	8	0.660	0.0825		

Table-S25: Leaf Sulfur

**Source**	**Df**	**Sum Sq**	**Mean Sq**	**F value**	**Pr(>F)**
**Treatment**	3	1.0667	0.3556	13.57	0.00167**
**Residuals**	8	0.2096	0.0262		

Table-S26: Root Nitrogen

**Source**	**Df**	**Sum Sq**	**Mean Sq**	**F value**	**Pr(>F)**
**Treatment**	3	6.082	2.0275	15.37	0.0011**
**Residuals**	8	1.055	0.1319		

Table-S27: Root Phosphorus

**Source**	**Df**	**Sum Sq**	**Mean Sq**	**F value**	**Pr(>F)**
**Treatment**	3	9.536	3.179	25.09	0.000201 ***
**Residuals**	8	1.013	0.127		

Table-S28: Root Potassium

**Source**	**Df**	**Sum Sq**	**Mean Sq**	**F value**	**Pr(>F)**
**Treatment**	3	0.7646	0.25486	6.737	0.014 *
**Residuals**	8	0.3026	0.03783		

Table-S29: Root Calcium

**Source**	**Df**	**Sum Sq**	**Mean Sq**	**F value**	**Pr(>F)**
**Treatment**	3	1.7583	0.5861	17.23	0.00075 ***
**Residuals**	8	0.2721	0.0340		

Table-S30: Root Magnesium

**Source**	**Df**	**Sum Sq**	**Mean Sq**	**F value**	**Pr(>F)**
**Treatment**	3	1.2155	0.4052	28.37	0.000129 ***
**Residuals**	8	0.1143	0.0143		

Table-S31: Root Sulfur

Source	Df	Sum Sq	Mean Sq	F value	Pr(>F)
**Treatment**	3	0.8338	0.27794	19.98	0.00045 ***
**Residuals**	8	0.1113	0.01391		

Table-S32: Root: Shoot Ratio

Source	Df	Sum Sq	Mean Sq	F value	Pr(>F)
**Treatment**	3	5.8734	1.9578	4.14	0.0481*
**Residuals**	8	3.7868	0.4734		
